# Stress distribution in pediatric zirconia crowns depending on different tooth preparation and cement type: a finite element analysis

**DOI:** 10.1186/s12903-022-02596-2

**Published:** 2022-12-01

**Authors:** Sang-Yeop Chung, Hyeonjong Lee, Yong Kwon Chae, Yun Sun Jung, Su-Sung Jo, Ko Eun Lee, Sung Chul Choi, Ok Hyung Nam

**Affiliations:** 1 Department of Civil and Environmental Engineering, Sejong University, Seoul, Korea; 2 Department of Prosthodontics, Dental College, Yonsei University, Seoul, Korea; 3 Department of Pediatric Dentistry, School of Dentistry, Kyung Hee University, Kyungheedae-Ro 26, Dongdaemoon-Gu, Seoul, 02447 Korea; 4 Present Address: Department of Pediatric Dentistry, Kyung Hee University College of Dentistry, Kyung Hee Universtiy Medical Center, Seoul, Korea

**Keywords:** Prefabricated zirconia crown, Primary teeth, Finite element analysis, Tooth preparation

## Abstract

**Background:**

In clinical settings, tooth preparation for prefabricated zirconia crowns (PZCs) in the primary dentition varies widely. However, knowledge about the biomechanical behavior of PZCs in various clinical settings is limited. This study was conducted to evaluate the biomechanical behavior of PZCs in different clinical settings using 3-dimensional finite element analysis.

**Methods:**

3-dimensional models of the PZC, cement, and tooth with six different conditions were simulated in primary molar teeth, incorporating cement thickness (100, 500, and 1000 μm) and cement type (resin-modified glass ionomer cement and resin cement). A total of 200 N of occlusal force was applied to the models, both vertically and obliquely as representative cases. A general linear model univariate analysis with partial eta-squared (ηp^2^) was performed to evaluate the relative effects of the variables.

**Results:**

The overall stress of tooth was increased as the cement space increases under oblique loading. The von Mises stress values of the resin cements were significantly higher than those of the resin-modified glass ionomer cements for all cement thicknesses (*p* < .05). The effect size of the cement type (η_p_^2^ = .519) was more dominant than the cement thickness (η_p_^2^ = .132) in the cement layer.

**Conclusions:**

Within the limits of this study, cement type has a greater influence on the biomechanical behavior of PZCs than cement thickness.

## Background

Pediatric zirconia crowns (PZCs) are currently available dental restorations for primary dentition with a highly reliable long-term success rate [1–3]. Retention of PZCs is affected by crown adaptation as well as adhesion between the tooth and the luting cement [4]. Tooth preparation can be aggressively performed to obtain the passive fit required by PZCs, resulting in reduced retention [5]. Wide variations in tooth preparation of PZCs were previously reported, and differences in tooth preparation forms were highlighted on occlusal surfaces [4]. Moreover, a laboratory study demonstrated that the remaining crown height after tooth preparation is strongly related to the retention of PZCs [6].

A recent systematic review showed that various types of luting materials, including glass ionomer (GI), resin-modified glass ionomer (RMGI), and resin cement, have been used in luting PZCs [7]. Within this review, failures in PZC retention (11%) were associated with luting cement materials. In addition, a previous clinical study reported that the retention of PZCs was influenced by cement materials during a 3-year follow-up period [8]. Despite the growing interest in luting cements for PZCs, the type of cement that exhibits superior performance due to the difference in the amount of tooth preparation has not been yet identified. This question is clinically relevant to pediatric dentists; if the amount of tooth preparation for PZCs is unavoidably excessive, the selection of luting cement is an important factor that clinicians can control.

The purpose of this study was to evaluate the biomechanical behavior of PZCs in different clinical settings, such as cement type and thickness and loading direction, using 3-dimensional (3D) simulation. Finite element analysis (FEA) is a tool used to visualize the mechanical behaviors of materials using interacting variables, and the stress and strain distributions of complex structures were examined using the obtained FEA results [9]. FEA has been previously used to investigate the biomechanical behaviors of dental materials [9–11]. The null hypothesis of this study was that there would not be different stress values of dental cements in PZCs depending on the amount of tooth preparation.

## Methods

### Generation of the geometric models

The geometric models were designed as previously described, with some modifications [4]. The mixed dentition dentiform model was digitally scanned (Identica T500; Medit Inc.). The scanned model was imported into a 3D modeling software (Meshmixer; Autodesk). The outer surfaces of the PZCs were aligned on the mixed dentition model, and the appropriate size of PZCs was determined to achieve proper mesiodistal contact with adjacent typodont teeth. Using 3D design software (GOM Inspect 2018 software; GOM GmbH), the outer surfaces of the PZCs were aligned at a position to achieve occlusal harmony with the marginal ridge of adjacent typodont teeth. The inner surfaces of the PZCs were then superimposed on the corresponding outer surfaces on the x-, y-, and z-axes. To simulate difference in tooth preparation, three layers of different thicknesses (100, 500, and 1000 μm) were evenly reduced from the inner surfaces and denoted as cement 100, cement 500, and cement 1000, respectively, indicating the cement thickness.

### Generation of finite element mesh

**Fig. 1:**
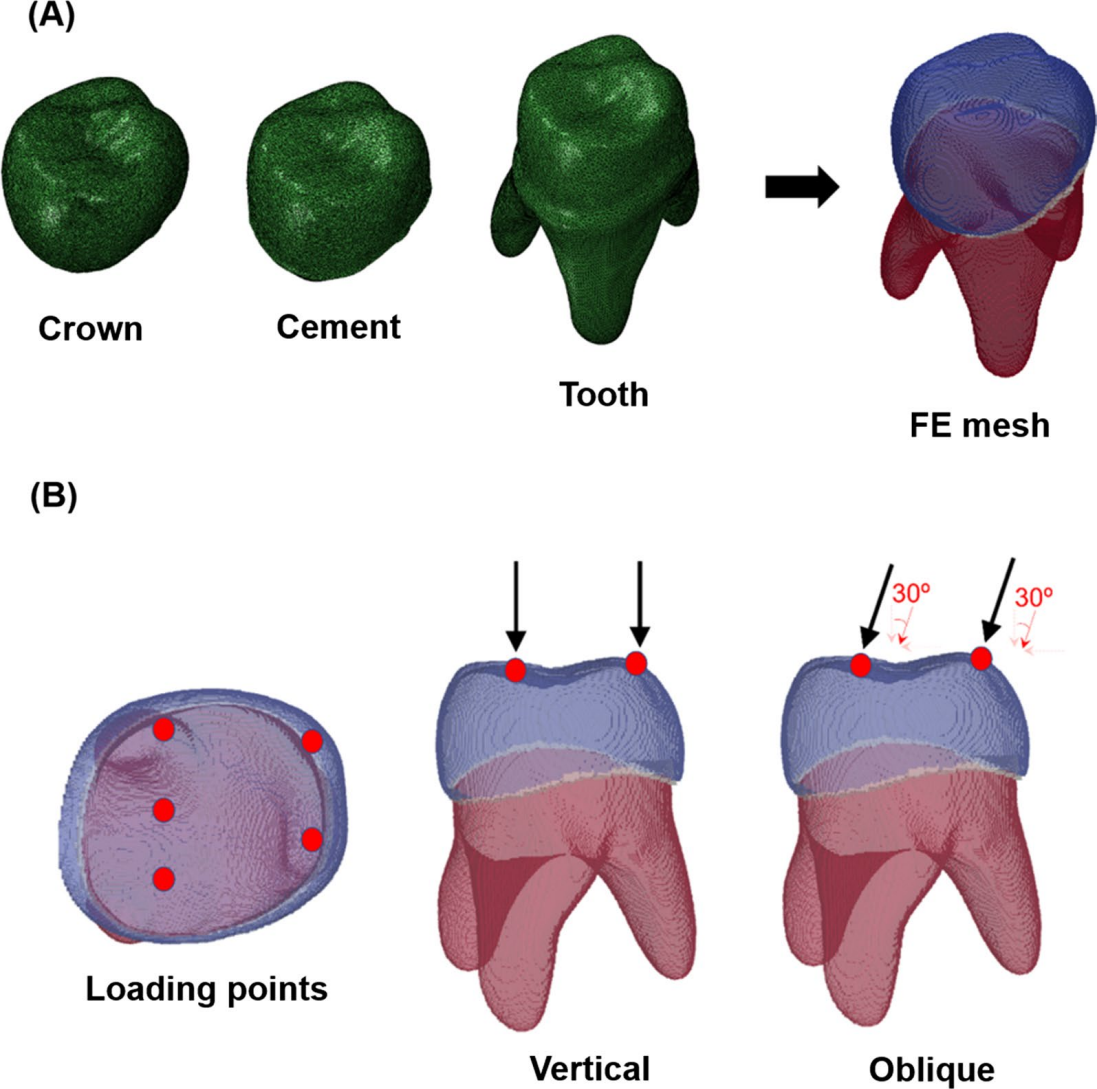
Establishment of finite element mesh model and loading conditions. **a** Geometric models of prefabricated zirconia crown, cement, and tooth part, and their combined finite element (FE) mesh model (cement 500 case). Note that blue, gray, and red parts in the FE mesh are crown, cement, and tooth, respectively. **b** Loading conditions for simulating mastication of children. For both vertical and angular forces, three points on the fossa and two points on the cusps were considered, and 40 N of force was applied to each node (200 N in total)

The geometric models were converted into a format for the FEA, as shown in Fig. 1a. In this study, a reliable commercial finite element package, the ABAQUS (CAE/2020, Dassault System, France), was used for the simulation, and all models were imported and meshed with eight-node brick (C3D8) elements to minimize the mesh dependency. The resolution of the voxels was 1 μm considering the computational cost. The properties of the materials used were selected from previous studies [12–16] and are listed in Table 1. The number of elements in each model was as follows:

(i) Cement 100: 126,346 elements in crown, 35,129 elements in cement, and 798,062 elements in tooth.
(ii) Cement 500: 126,357 elements in crown, 89,609 elements in cement, and 686,875 elements in tooth.
(iii) Cement 1000: 126,344 elements in crown, 172,168 elements in cement, and 571,023 elements in tooth.

**Table 1:** Material properties for the finite element analysis

Component	Material	Elastic modulus (GPa)	Poisson’s ratio	Compressive strength (MPa)
Crown	Monolithic zirconia	205	0.19	1000
Cement	RMGI	3.7	0.30	210
	Resin	8.9	0.35	291
Tooth	Dentin	19.89	0.31	297

### Applied forces and their environment

In the FEA, all components, including the crown, cement, and tooth, were assumed to be homogeneous and perfectly bonded without marginal gaps, while considering slip. To describe a realistic phenomenon, the exposed root surfaces of the tooth were fully constrained for all the degrees of freedom.

To investigate the mechanical behavior of the models according to masticatory movement, two types of forces in different directions were considered representative: vertical and oblique (30°). As shown in Fig. 1b, five different points on occlusal surfaces were selected for loading, including three points on the fossa and two points on the cusp. These points were assumed to be contact areas during mastication. A magnitude of 40 N per node was applied (200 N in total) to simulate mastication of children with primary dentition [17]. For the vertical case, the force was applied in the perpendicular direction of the crown, and oblique force was applied along the 30° inclined direction from the vertical direction. A static load was applied to all models, and the von Mises stress and maximum principal strains of all elements were examined to compare the effect of the cement thickness and type on the tooth behavior.

### Statistical analysis

Data were analyzed using IBM SPSS Statistics 20 (SPSS Inc., Chicago, IL, USA) and GraphPad Prism 9.3.1 (GraphPad Software Inc., San Diego, CA, USA). To compare stress values between RMGI and resin, ANOVA test with Tukey HSD test as post hoc analysis was performed after 50 values were randomly selected among the top 5% values of the element von Mises stress as previously described with some modifications [18]. A general linear model univariate analysis with partial eta-squared (η_p_^2^) was performed to evaluate the relative effects of independent variables (force direction, cement thickness, and cement type) and their interactions [18, 19]. *P* values < 0.05 were considered statistically significant.

## Results

**Fig. 2:**
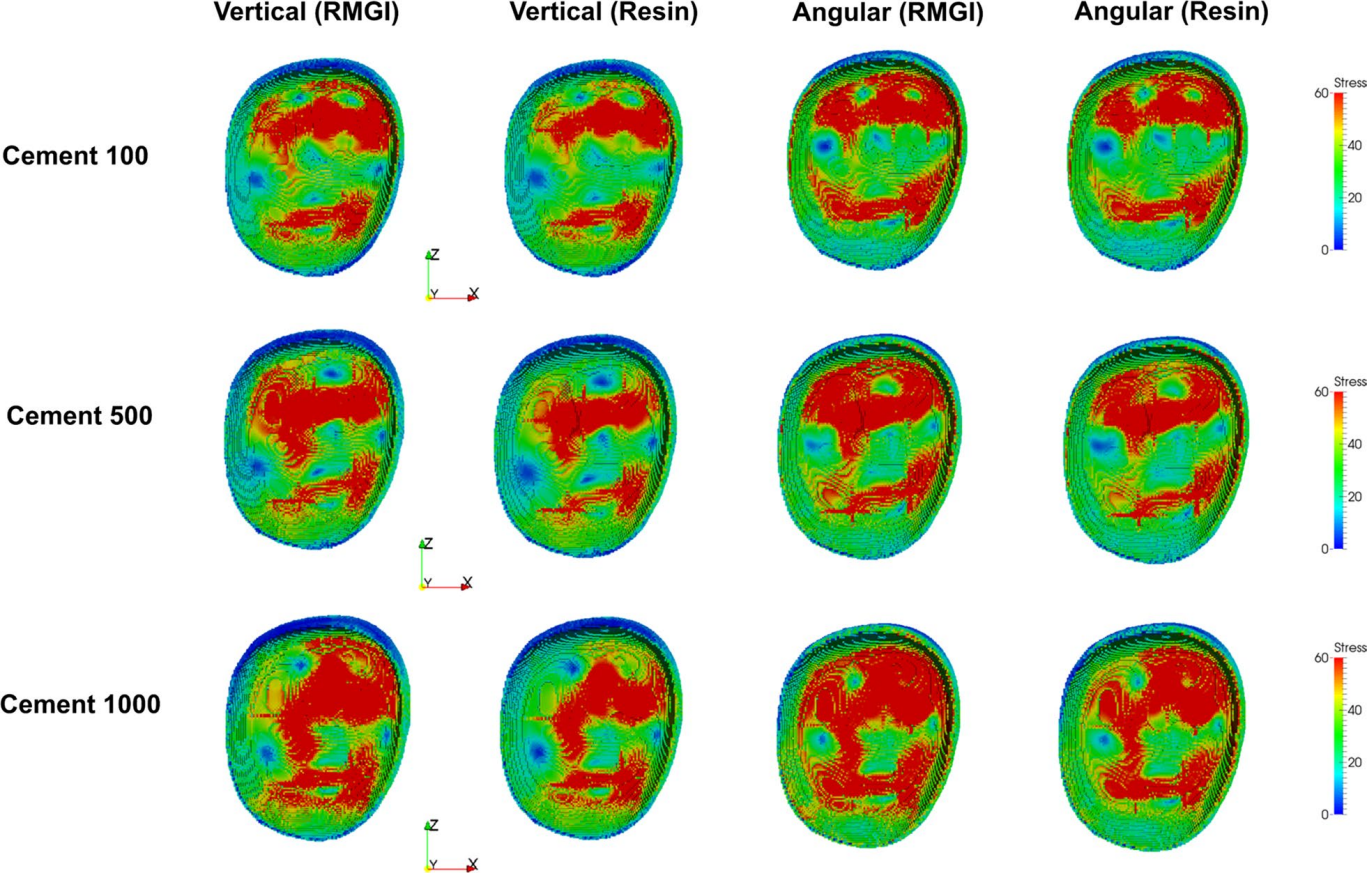
Stress distribution (MPa) at crown level for each model. Stress level was presented as color-coded map. Red color represents to high stress concentration and blue color represents less stress concentration

**Fig. 3:**
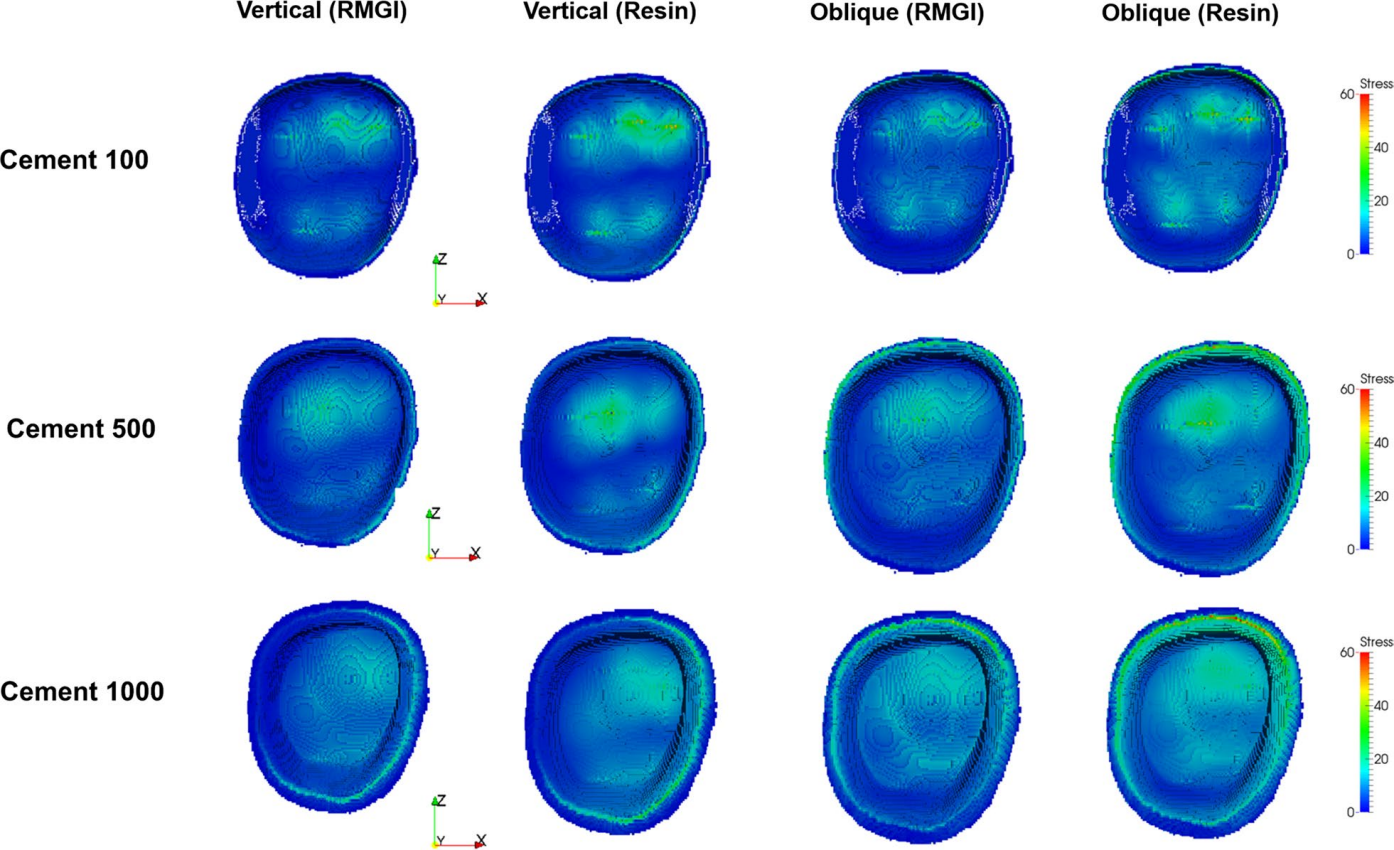
Stress distribution (MPa) at cement level for each model. Stress level was presented as color-coded map. Red color represents to high stress concentration and blue color represents less stress concentration

**Fig. 4:**
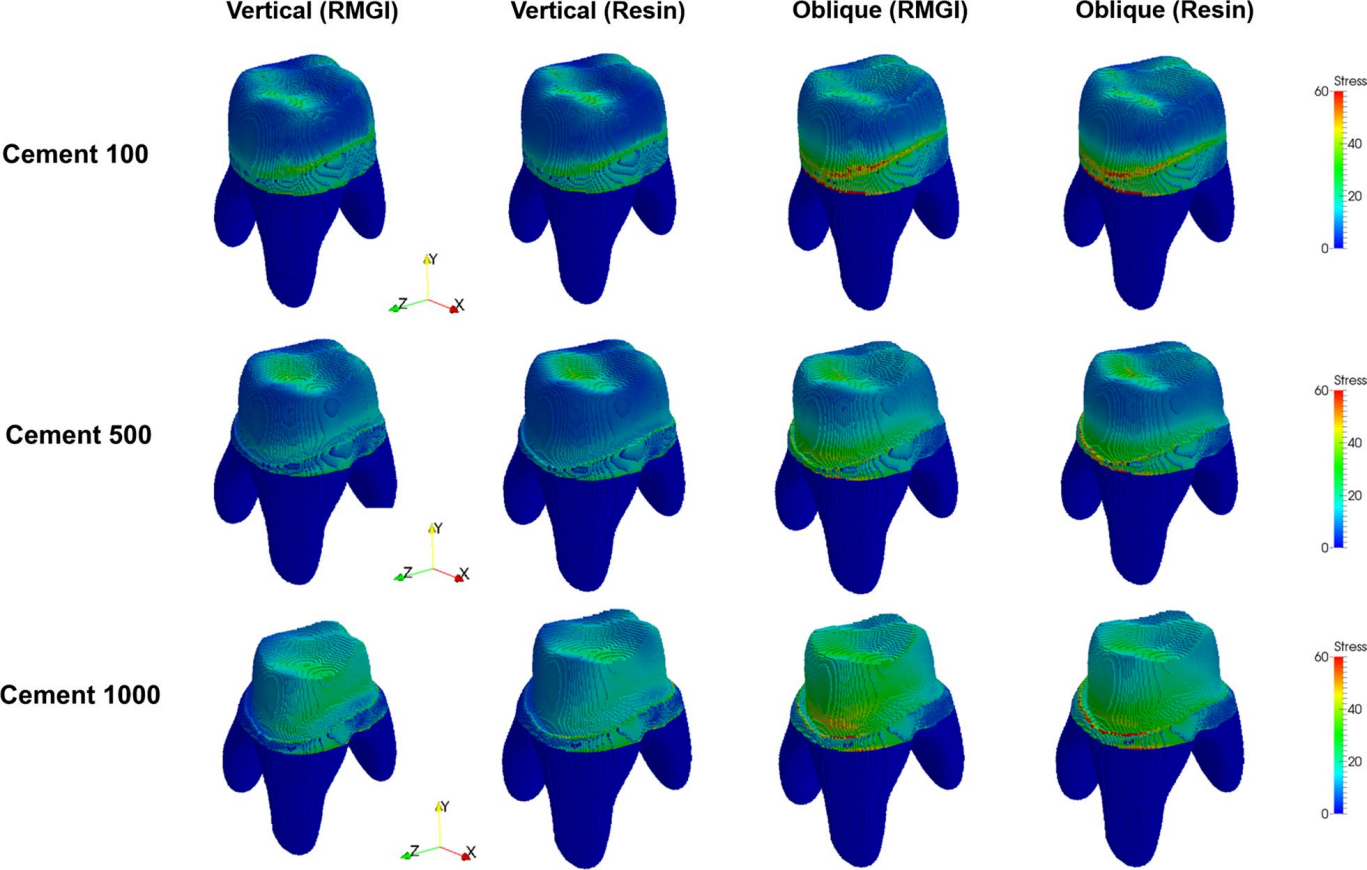
Stress distribution (MPa) at tooth level for each model. Stress level was presented as color-coded map. Red color represents high stress concentration and blue color represents less stress concentration

Figures 2, 3, and 4 illustrate the stress distribution according to the cement thickness model and luting cement. Stress was observed in greater values of the cement space. This trend was highlighted when an oblique force was applied.

**Fig. 5:**
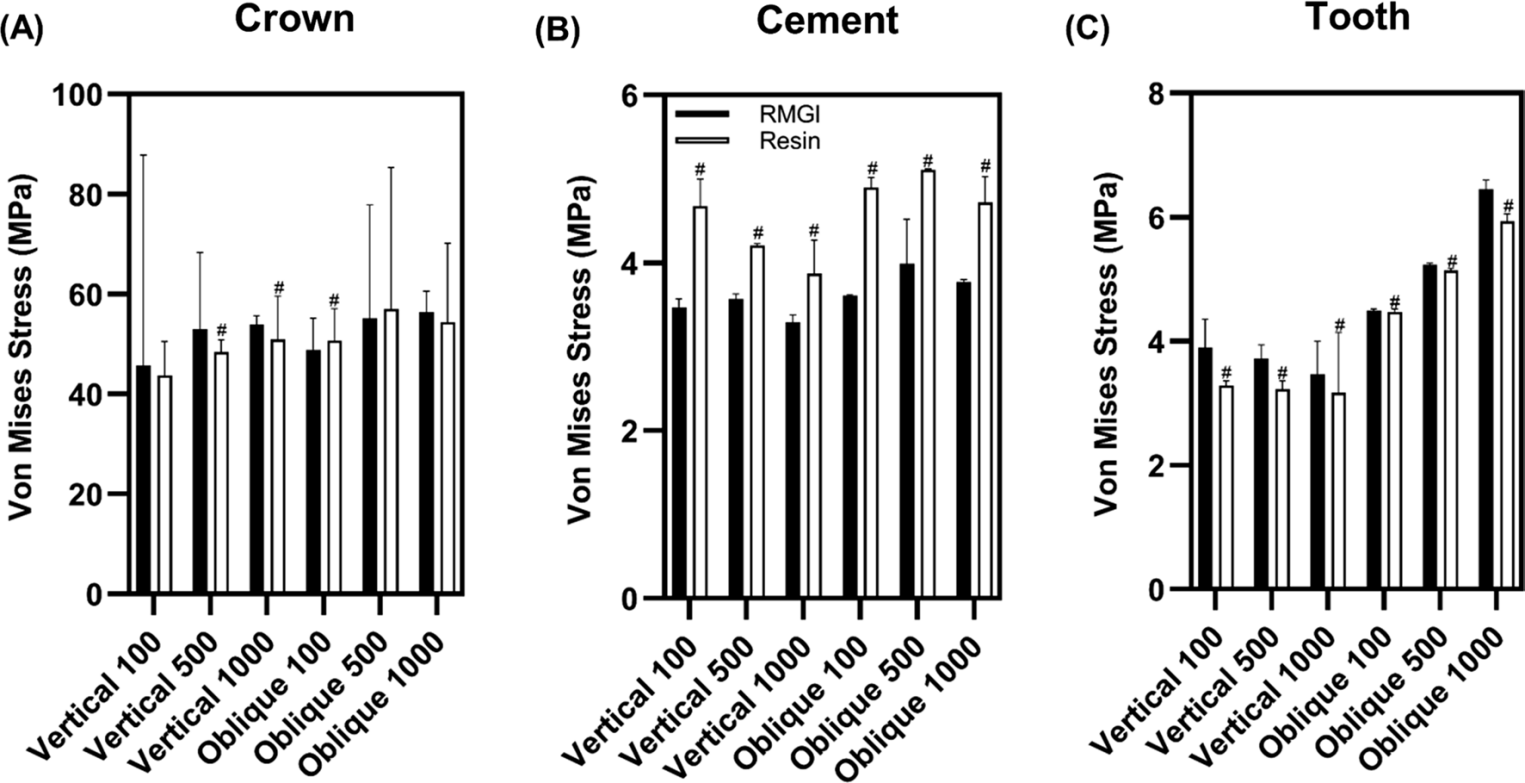
von Mises stress values (MPa) in each model according to loading conditions. **a** Crown layer, **b** cement layer, **c** tooth layer. Within vertical and oblique forces, different case letters represent statistically significant (*p* < .05)

A comparison of the von Mises stress values for each model is shown in Fig. 5. In the crown layer, there were no significant differences among the different loading conditions. In the cement layer, a significantly higher stress value was observed in the resin than in the RMGI in all the cement thickness models (*p* < 0.05). Interestingly, greater stress values were not seen with increasing tooth preparation in each cement group. In the tooth layer, higher values were observed for RMGI than for resin (*p* < 0.05). Greater stress was associated with greater tooth preparation in each cement group.

The general linear model univariate analysis revealed that von Mises stress was significantly associated with the direction of the external force, cement layer thickness, and cement type (Table 2). On the crown layer, von Mises stress was significantly associated with force direction, cement thickness, and cement type. The effect size was the highest for the cement thickness (η_p_^2^ = 0.018) in 1-way analysis. However, there was no interaction on stress between 2-way and 3-way interactions except for interactions with force direction and cement thickness (*p* > 0.05). Regarding the cement layer, the stress was significantly different depending on the force direction, cement thickness, and cement type (*p* < 0.001). The stress in the cement layer was strongly affected by cement type (η_p_^2^ = 0.519). In the tooth layer, stress was significantly associated with the force direction, cement thickness, and cement type (*p* < 0.0001). The effect size of the cement thickness (η_p_^2^ = 0.890) was higher than that of the cement type (η_p_^2^ = 0.01).

**Table 2:** Effect size of factors on von Mises stress

Factors	Crown		Cement		Tooth	
	*p *value	Effect size^a^	*p *value	Effect size	*p *value	Effect size
*1-way*						
Force direction	< .0001	.007	< .0001	.231	< .0001	.890
Cement thickness	< .0001	.018	< .0001	.132	< .0001	.792
Cement type	< .0001	.001	< .0001	.519	< .0001	.01
*2-way*						
Force direction × Cement thickness	< .0001	.001	< .0001	.036	< .0001	.367
Force direction × Cement type	.128	–	< .001	.026	< .0001	.083
Cement thickness × Cement type	.420	–	< .001	.024	< .0001	.157
*3-way*						
Force direction × Cement thickness × Cement type	.943	-	< .0001	.007	< .0001	.002

^a^Effect size = Partial eta squared (η_p_^2^)

**Fig. 6:**
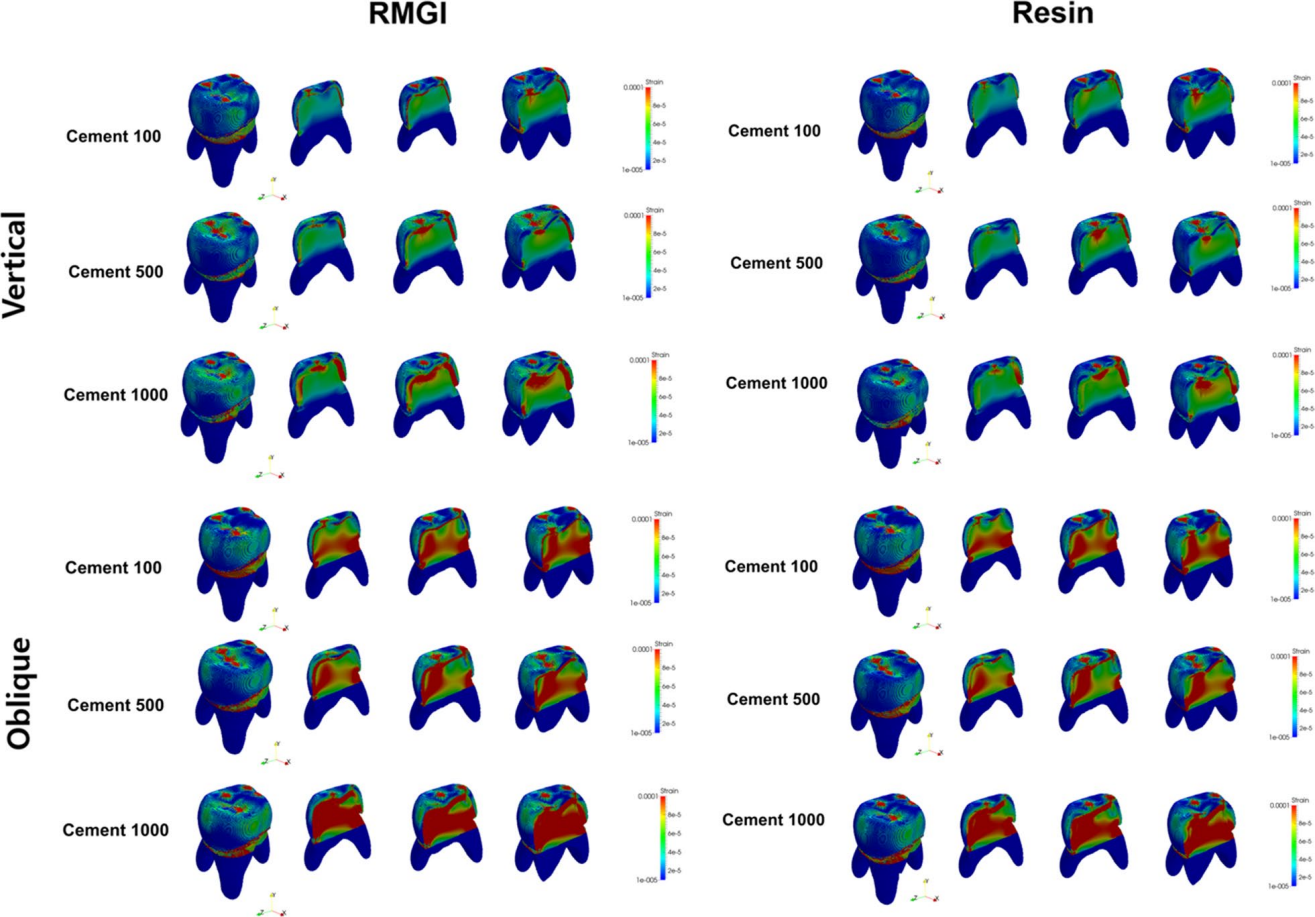
Strain distribution of whole layers in each model. Strain level was presented as color-coded map. Red color represents high stress concentration and blue color represents less stress concentration

The strain distribution of each model in the whole components, which can describe the displacement-related characteristic, is presented in Fig. 6. The strain concentration was highlighted in the cement layer in all models. In both cement material models, relatively large strains were found in the case of the oblique force, particularly in the Cement 1000 case. In the case of the vertical force, the strain concentration existed mainly in the side of the cement region, while the region with large strain was found in both overall cement and inside of the tooth.

## Discussion

As the crowns for primary teeth are prefabricated, pediatric dentists must pay careful attention to crown adaptation and cementation. In addition, there are wide variations in the degree of PZC adaptation by tooth preparation in clinical situations [4]. Thus, the evaluation of the biomechanical behavior of PZCs in various situations is of clinical significance.

This study evaluated the stress distribution of PZCs. Although the estimated stress on crowns varied depending on cement thickness and cement type, the stress values were below the yield strength of a typical zirconia crown [20]. Likewise, stress exerted on teeth would not hinder luting considering the elastic moduli of enamel and dentin, which are approximately 80 GPa and 20 GPa, respectively [21]. Regardless of the direction of occlusal force, the stress values on the PZC and tooth were within the limit of yield strength or elastic modulus of teeth, cement, and zirconia crown. Thus, PZCs can withstand a normal occlusal bite force in children.

Within a normal occlusal bite force in children, the stress concentration occurred in the vicinity of the points where the forces were applied in the crown and cement layer. Especially, more regions with large stress intensity were observed in case of oblique force than those of the vertical force. This denotes oblique force can be more critical than the vertical force. Concerning the cement types, the tooth with RMGI cement tends to have larger stress intensity and more regions with relatively large stress values, and this indicates that RMGI can be more vulnerable to external load regardless of the cement layers and force directions.

In this study, the estimated stress values on the cement layer were 3–5 MPa (Fig. 5b). GI cements have commonly been used in luting PZCs [7]. However, GI cement showed 2.6 MPa of shear bond strength to zirconia block in a previous study [22]. Another laboratory study reported that shear bond strength of GI cements to PZCs was 4.23 MPa after thermocycling [23]. Based on these findings, GI cements may not to be sufficient for luting PZCs. Previous studies have reported that the shear bond strength of RMGI cement is 4–16 MPa [22, 24–26]. Although the stress values of RMGI cements ranged from 3 to 4 MPa, there may be a risk of cohesive failure between PZC and RMGI cements because vertical and oblique forces interact in the oral cavity. On the other hand, resin cement was reported to have 30–400% higher shear bond strength than RMGI cements [22, 26, 27]. Although the distributed stress values were significantly higher on resin cement than RMGI in this study, the stresses exerted on resin cements were within shear bond strength of resin cement. Thus, at least in terms of mechanical properties, resin cements are considered more advantageous for PZC retention. However, the handling of resin cements requires more caution compared to other types of cements for PZCs. As PZCs are hardly translucent, adequate curing time is advisable to ensure a proper degree of conversion of the resin cement [28]. Also, excess resin cement is difficult to remove after cementation [29].

Unlike conventional fixed prostheses, it is difficult to ensure a precise fit between PZCs and primary teeth, resulting in increased cement thickness. Pediatric dentists may express concern about providing adequate PZC retention in clinical situations. Interestingly, in this study, the difference in stress distribution was not remarkably obvious according to the cement thickness up to 1000 μm. The Cement 100 model was designed to simulate the ideal conditions of PZCs, because approximately 100 μm of cement thickness is considered clinically acceptable for zirconia crowns in permanent teeth [30]. This finding suggests that the cement layer would not become mechanically weak unless extensive tooth reduction was performed. In addition, this finding was supported by the general linear model univariate analysis. In this study, the effect size of cement thickness was 0.132, whereas that of cement type was 0.519. The analysis demonstrated that the cement type was the predominant factor in determining the stress on the cement layer with respect to the cement thickness. Thus, there may be a limited effect on PZC retention according to the amount of tooth preparation, unless extremely extensive tooth reduction is performed. In clinical situations, however, pediatric dentists should prepare teeth for PZCs with caution because extensive preparation will result in smaller surface areas for cementation. A previous study recommended an occlusocervical height greater than 2 mm for PZCs in primary molars [6].

As confirmed from the obtained results, the effect of different conditions, such as PZC and cement type, on the mechanical behavior of primary teeth can be effectively described and compared using FEA. Because this study was conducted using FEA, the modelled or discretized PZCs and cements do not exactly replicate real PZCs and luting cements. Therefore, further in vitro studies should be continued to improve the reliability of the results of this study.

Furthermore, this study was conducted using only one tooth type for the analysis. Therefore, a limitation in the present study is that the results do not precisely reflect the conditions of real PZCs and cements. In addition, because it is practically impossible to prepare teeth with uniform thickness in the clinic, the difference in the amount of preparation may be a potential variable affecting the retention of PZCs; however, this factor was not accounted for in this study. Despite these limitations, this study was the first to evaluate the biomechanical behavior of PZCs depending on tooth preparation and cement type, and the results demonstrated comparable effects of different conditions, which can be further confirmed in detail by incorporating experiments This study is significant in that it is a structural-mechanical analysis that suggests that selection of proper luting cement is beneficial to PZCs.

## Conclusions

In conclusion, the biomechanical behavior of PZCs under variable loading conditions was evaluated using FEA. Within the limits of this study, it was confirmed that cement type is more important in determining the biomechanical behavior of PZCs than tooth preparation, such as cement thickness. It was also demonstrated that mechanical properties of resin cement contribute to stress distribution between PZCs and the teeth regardless of amounts of tooth preparation.

## Data Availability

All data generated or analyzed from this study are included in this published article.

## Abbreviations

PZC: Prefabricated zirconia crowns
GI: Glass ionomer
RMGI: Resin-modified glass ionomer
3D: 3-Dimensional
FEA: Finite element analysis
